# Short-duration chemoprophylaxis might reduce incidence of deep vein thrombosis in Asian patients undergoing total knee arthroplasty

**DOI:** 10.1186/s43019-020-00077-w

**Published:** 2020-11-04

**Authors:** Siyuan Zhang, Kway Swar Htet, Xin Yang Tan, Xinyu Wang, Wilson Wang, Weiliang Chua

**Affiliations:** 1 Yong Loo Lin School of Medicine, National University of Singapore, 10 Medical Drive, Singapore, 117597 Singapore; 2 Department of Orthopaedic Surgery, National University Hospital, 1E Kent Ridge Road, Singapore, 119228 Singapore

**Keywords:** Deep vein thrombosis, Venous thromboembolism, Total knee arthroplasty, Total knee replacement, Chemoprophylaxis

## Abstract

**Background:**

Venous thromboembolism (VTE) is a serious complication that may occur after total knee arthroplasty (TKA), leading to the recommendation of routine chemoprophylaxis by international guidelines. This study aims to determine if short-duration chemoprophylaxis after TKA reduces the incidence of VTE in an Asian population.

**Methods:**

A retrospective study of 316 patients who underwent unilateral primary TKA between 1 January 2011 and 31 December 2013 was conducted. All patients received mechanical prophylaxis. One hundred seventeen patients (37%) received additional chemoprophylaxis, whereas 199 patients (63%) did not. A Doppler ultrasound (DUS) of both lower limbs was conducted for all patients within 6 days after surgery (median = 3 days) to assess for both proximal and distal DVT. Chemoprophylaxis in the form of enoxaparin (low molecular weight heparin; LMWH), aspirin, or heparin was administered until patients had a normal DUS, for a median duration of 4 days. Patients were followed up clinically for a minimum of 6 months to monitor for delayed or recurrent VTE and at least 2 years for patient-reported outcome measures.

**Results:**

Overall, 24 patients (7.59%) developed deep vein thrombosis (DVT): three proximal and 21 distal DVTs. Twenty-three of the 24 patients were asymptomatic. Twenty of 199 patients (10.05%) with only mechanical prophylaxis developed DVT, whereas four of 117 patients (3.42%) with additional chemoprophylaxis developed DVT. Multivariate analysis showed that chemoprophylaxis use was associated with reduced incidence of DVT (odds ratio = 0.19, *p* value = 0.011). Other factors associated with increased DVT incidence include female gender (odds ratio = 5.45, *p* value = 0.034), positive history of cancer (odds ratio = 5.14, *p* value = 0.044), and increased length of stay in hospital (odds ratio = 1.19, *p* value < 0.001).

**Conclusions:**

Our study has shown that despite the low incidence of DVT in Asian patients undergoing TKA, short-duration chemoprophylaxis might be effective in reducing the incidence of DVT. However, most DVTs observed in our study were distal and may be of limited clinical significance. Further studies are needed to investigate the impact of chemoprophylaxis use on the incidence of PE and overall mortality rates among Asian patients.

## Introduction

Total knee arthroplasty (TKA) is a widely used procedure to help patients with symptomatic, end-stage knee osteoarthritis relieve pain and improve function [1]. Patients undergoing TKA are at risk for developing venous thromboembolism (VTE), which includes deep vein thrombosis (DVT) and pulmonary embolism (PE) [2]. Data from Western populations have suggested that, in patients undergoing total joint arthroplasty without chemoprophylaxis, the rates of DVT can be as high as 35–84% [3].

Western guidelines recommend routine thromboprophylaxis for patients undergoing TKA. The American Academy of Orthopaedic Surgeons (AAOS) and the United Kingdom’s National Institute for Health and Care Excellence (NICE) both recommend routine pharmacological thromboprophylaxis for those patients undergoing elective knee replacement surgery whose VTE risk outweighs their risk of bleeding [4, 5].

However, a lack of consensus remains regarding the use of routine chemoprophylaxis in Asia. Mechanical thromboprophylaxis, early postoperative mobilization, and shorter hospital stays have led some to question whether routine chemoprophylaxis is still necessary [6, 7]. Moreover, the incidence of VTE among Asians is generally thought to be lower compared to their Western counterparts [8–11]. In a systematic review of Asian patients undergoing TKA without thromboprophylaxis, Kanchanabat et al., using venography, found that the incidence of proximal DVT, distal DVT, and symptomatic PE was 8.7%, 30.0%, and 0.5%, respectively [8]. Using Doppler ultrasound (DUS), Loh et al. and Won et al. also reported overall VTE rates of 4.6% and 4.3%, respectively, in Asians undergoing TKA without chemical thromboprophylaxis [10, 12]. These findings raise a debate about whether the potential risks of chemoprophylaxis, such as bleeding and increased transfusion rates, outweigh its potential benefits in Asian patients [13, 14].

The primary aim of this study is to determine if the use of chemoprophylaxis in addition to mechanical prophylaxis after primary TKA reduces the incidence of postoperative VTE in an Asian population. The secondary aim is to identify risk factors associated with increased incidence of thromboembolism after TKA. Our hypothesis is that additional chemoprophylaxis is effective in reducing the incidence of VTE after TKA.

## Methods

We conducted a retrospective study of all patients who underwent elective primary TKA for knee osteoarthritis at an academic tertiary hospital in Singapore between 1 January 2011 and 31 December 2013. Ethics approval was obtained from the Institutional Review Board. Data was collected from both electronic and paper records.

Inclusion criteria were all patients who underwent elective primary TKA during the specified period. Surgeries were performed under general or spinal anesthesia. All TKAs were performed with tourniquet control and without tranexamic acid. Patients who had unicompartmental knee arthroplasty, bilateral simultaneous TKA, and revision TKAs were excluded from the study.

### Postoperative mobilization and VTE prophylaxis protocol

Patients were allowed to stand from postoperative day (POD) 1 and progressed to full weight-bearing activity with walking aids as tolerated. Patients also received physiotherapy and occupational therapy daily for postoperative mobilization and rehabilitation until they were discharged.

All 316 patients were started on mechanical thromboprophylaxis immediately postoperatively in the form of intermittent pneumatic compression pumps (Arjo Huntleigh Flowtron Excel® DVT pump system). Mechanical thromboprophylaxis was continued until patients were discharged from the hospital (median = 6 days). The decision to administer chemoprophylaxis in the form of enoxaparin (low molecular weight heparin; LMWH) or aspirin was based on the surgeon’s preference and the patient’s VTE and bleeding risks. One hundred seventeen patients received chemoprophylaxis (median 4 days), most patients (113) received enoxaparin (30 or 40 mg according to patient weight), three received aspirin (150 mg), and one received heparin.

All patients underwent a DUS of both lower limbs within 6 days (median = 3 days) after their operations to detect the presence of DVT (as per hospital protocol). Short-duration, in-hospital-only chemoprophylaxis was given until DUS of both lower limbs confirmed an absence of DVT (median = 4 days). All patients were followed up clinically for a minimum of 6 months postoperatively to monitor for symptoms of delayed or recurrent VTE. Repeat DUS would have been performed if a clinical suspicion of DVT had occurred.

For patients found to have VTE, management and treatment (if any) would be decided after consultation with a hematologist. These patients would then be followed up by hematology for further VTE management.

### Data collection

Data collected include patient demographics such as age, gender, and body mass index (BMI); significant comorbidities such as hypertension, ischemic heart disease, previous VTE, and history of cancer; and surgical factors such as duration of surgery, length of stay, and surgical complications. The presence or absence of chemoprophylaxis use, as well as the presence or absence of VTE, was also recorded.

The presence of DVT was assessed via Doppler Ultrasound (DUS) that was conducted within 6 days after surgery (median = POD3). DUS is the imaging modality in our hospital protocol, as it is noninvasive with no radiation risk. The presence of DVT was defined as the lack of compressibility and impedance of normal blood flow in the affected veins. The trifurcation point of the popliteal vein was used as the demarcation between proximal and distal DVT. Patients who had symptoms suggestive of PE underwent computed tomography pulmonary angiography (CTPA) for confirmation of PE.

Patients were followed up for at least 6 months postoperatively to monitor for delayed or recurrent VTE and 2 years postoperatively for patient-reported outcome measures (PROM). The Western Ontario and McMaster Universities Osteoarthritis Index (WOMAC), Short Form-36 (SF-36), and Knee Society Score (KSS) were used to objectively measure patients’ function and wellbeing pre-operatively, 6 months postoperatively, 12 months postoperatively, and 24 months postoperatively.

### Statistical analysis

All analysis was performed using R software version 3.6.1 (R Foundation for Statistical Computing, Vienna, Austria; 2019). Bivariate analysis was performed to describe the presence of DVT relative to patient demographics, significant comorbidities, and surgical factors, as well as to the use of chemoprophylaxis. Pearson’s chi-square test was used for categorical variables, and Mann-Whitney’s U test was used for continuous variables.

This was followed by multivariate analysis of factors associated with DVT incidence. All variables with a *p* value of less than 0.1 in bivariate analysis were included in the multiple logistic regression model. Bivariate analysis was also performed to describe chemoprophylaxis use relative to postoperative bleeding complications and PROM.

## Results

A total of 316 patients were included in this retrospective study, including 89 men and 227 women. The mean age was 62.3 years (range: 41–88), mean body mass index (BMI) was 28.7 (range: 16.6–51.1), mean duration of surgery was 106.6 min (range: 60–237), and median length of stay was 6 days (range: 4–36).

All patients received mechanical thromboprophylaxis postoperatively. One hundred seventeen patients (37%) received chemoprophylaxis, whereas 199 patients (63%) did not receive any chemoprophylaxis. Of the 117 patients who received chemoprophylaxis, 113 received enoxaparin (LMWH), three received aspirin, and one received heparin.

Baseline patient characteristics can be seen in Table 1, and bivariate and multivariate analysis of factors associated with DVT incidence can be seen in Table 2. Significant differences in age, obesity, and presence of ischemic heart disease were observed between patients who were and were not given chemoprophylaxis. These factors were included in multivariate analysis of factors related to DVT incidence to adjust for potential confounding effects (Table 2).

**Table 1:** Baseline patient characteristics relative to chemoprophylaxis use

Variable	Overall (*n* = 316)	Given chemoprophylaxis		*p* value
		Yes (*n* = 117)	No (*n* = 199)	
Age (years)				< 0.001***
Mean	65.3	67.5	63.9	
Median	65.0	67.0	64.0	
SD	8.5	8.5	8.3	
Range	41–88	47–88	41–83	
Gender				0.596
Female	227 (71.8%)	82 (70.1%)	145 (72.9%)	
Male	89 (28.2%)	35 (29.9%)	54 (27.1%)	
BMI				0.016*
Mean	28.7	27.7	29.3	
Median	28.6	27.5	29.0	
SD	5.6	4.8	5.9	
Range	16.6–51.1	18.30–51.10	16.60–45.20	
Obesity (BMI > 30)				< 0.001***
Yes	101 (34.6%)	23 (21.3%)	78 (42.4%)	
No	191 (65.4%)	85 (78.7%)	106 (57.6%)	
Hypertension				0.821
Yes	197 (62.3%)	72 (61.5%)	125 (62.8%)	
No	119 (37.7%)	45 (38.5%)	74 (37.2%)	
Hyperlipidemia				0.402
Yes	174 (55.1%)	68 (58.1%)	106 (53.3%)	
No	142 (44.9%)	49 (41.9%)	93 (46.7%)	
Diabetes mellitus				0.382
Yes	79 (25.0%)	26 (22.2%)	53 (26.6%)	
No	237 (75.0%)	91 (77.8%)	146 (73.4%)	
Ischemic heart disease				< 0.001***
Yes	42 (13.3%)	28 (23.9%)	14 (7.0%)	
No	274 (86.7%)	89 (76.1%)	185 (93.0%)	
History of cancer				0.787
Yes	12 (3.8%)	4 (3.4%)	8 (4.0%)	
No	304 (96.2%)	113 (96.6%)	191 (96.0%)	

* denotes *p* value < 0.05, ** denotes *p* value < 0.01, *** denotes *p* value < 0.001

**Table 2:** Patient demographics, co-morbidities, and surgical factors relative to DVT incidence after TKA

Variable	Presence of DVT		Odds ratio	*p* value	Adjusted odds ratio	Adjusted *p* value
	Yes (*n* = 24)	No (*n* = 292)				
Age (years)				0.574	1.00 (0.95–1.06)	0.946
Mean	66.0	65.2				
Median	67.5	65.0				
SD	8.6	8.5				
Range	49–81	41–88				
Gender			2.92 (0.85–10.05)	0.076	5.45 (1.37–3.41)	0.034*
Female	21 (87.5%)	206 (70.5%)				
Male	3 (12.5%)	86 (29.5%)				
BMI				0.356		
Mean	29.2	28.6				
Median	29.3	28.0				
SD	4.7	5.7				
Range	18.8–36.5	16.6–51.1				
Obesity (BMI > 30)			1.39 (0.59–3.25)	0.447	0.87 (0.31–2.34)	0.989
Yes	10 (41.7%)	91 (34.0%)				
No	14 (58.3%)	177 (66.0%)				
Hypertension			0.83 (0.36–1.94)	0.673		
Yes	14 (58.3%)	183 (62.7%)				
No	10 (41.7%)	109 (37.3%)				
Hyperlipidemia			1.15 (0.50–2.69)	0.738		
Yes	14 (58.3%)	160 (54.8%)				
No	10 (41.7%)	132 (45.2%)				
Diabetes mellitus			1.00 (0.38–2.61)	0.999		
Yes	6 (25.0%)	73 (25.0%)				
No	18 (75.0%)	219 (75.0%)				
Ischemic heart disease			0.93 (0.26–3.25)	0.905	1.61 (0.23–8.33)	0.587
Yes	3 (12.5%)	39 (13.4%)				
No	21 (87.5%)	253 (86.6%)				
History of cancer			4.49 (1.13–17.85)	0.020*	5.14 (0.92–24.5)	0.044*
Yes	3 (12.5%)	9 (3.1%)				
No	21 (87.5%)	283 (96.9%)				
Surgical duration (min)				0.748		
Mean	106.0	106.6				
Median	111	104				
SD	18.4	20.7				
Range	72–140	60–237				
Length of stay (days)				< 0.001***	1.19 (1.08–1.31)	< 0.001***
Mean	10.2	7.2				
Median	8	6				
SD	6.5	3.4				
Range	4–36	4–28				
Chemoprophylaxis use			0.32 (0.11–0.95)	0.032*	0.19 (0.04–0.61)	0.011*
Yes	4 (16.7%)	113 (38.7%)				
No	20 (83.3%)	179 (61.3%)				

* denotes *p* value < 0.05, ** denotes *p* value < 0.1, *** denotes *p* value < 0.001

### Overall VTE rates and other complications

Of the 316 patients, 24 patients (7.59%) developed DVT: three proximal DVTs (0.95%) and 21 distal DVTs (6.65%). One DVT was symptomatic (4.17%), and 23 DVTs were asymptomatic (95.8%). Table 3 shows the type and location of the DVTs detected in our study.

**Table 3:** Type and location of DVT

Site			Number of patients
Total DVT			24
	Proximal DVT		3 (12.5%)
		Popliteal vein	3
	Distal DVT		21 (87.5%)
		Peroneal vein	9
		Posterior tibial vein	7
		Peroneal and posterior tibial vein	5

On early postoperative DUS scans (POD3–4), 22 patients were found to have DVT (two proximal and 20 distal). All 22 DVTs were asymptomatic. However, two additional patients with normal initial DUS scans were subsequently found to have DVT on repeat scans.

The first was a 79-year-old female with a normal DUS scan on POD4, who subsequently developed right lower limb swelling. A repeat scan on POD11 found a right distal (posterior tibial vein) DVT. No further anticoagulation was administered, and the patient was discharged while well on POD18, with subsequent normal scans. The second was a 57-year-old male with Factor V Leiden and prior history of VTE, on long-term warfarin therapy. He was treated perioperatively with enoxaparin, and warfarin was restarted after a normal DUS scan on POD4. He subsequently developed hemarthrosis requiring inpatient monitoring and a repeat DUS scan on POD18 found a proximal (popliteal vein) DVT. He was subsequently discharged while well, with no other complications on POD35.

In addition, a 70-year-old female who had not received chemoprophylaxis was found to have a distal DVT via DUS on POD3 and subsequently developed symptomatic PE diagnosed by CTPA on POD4. The patient was treated with enoxaparin and subsequently discharged while well on POD7.

All patients were clinically followed up for 6 months postoperatively to monitor for symptoms of delayed or recurrent VTE. No delayed VTE was diagnosed after discharge, nor were any other complications related to the use of chemoprophylaxis.

Treatment of patients with DVT was decided after consultation with hematologists and in view of the patient’s individual risk profile. All patients with proximal DVT (3) were treated with enoxaparin. Of the 21 patients with distal DVT, eight were treated with enoxaparin, and one was treated with aspirin, whereas the remaining 12 were managed with monitoring and repeat DUS scans.

### Subgroup analysis: patients with DVT

Of the 24 patients, three were male and 21 were female. The mean age was 66.0 years (range: 49–81), and the mean BMI was 29.2 (range: 18.8–36.5). The mean duration of surgery was 106.0 min (range: 72–140), and the median length of stay was 8 days (range: 4–36). Of these 24 patients who developed DVT, four had received chemoprophylaxis, whereas 20 had not.

### Factors associated with DVT incidence

Based on bivariate analysis (see Table 2), gender (*p* value = 0.076), history of cancer (*p* value = 0.020), length of stay (*p* value < 0.001), and chemoprophylaxis use (*p* value = 0.032) were identified as potentially significant variables. All variables with *p* value less than 0.1 were included in multivariate logistic regression to investigate their relation with DVT incidence. Age, obesity, and presence of ischemic heart disease were also included in multivariate analysis to adjust for potential confounding effects.

Multivariate analysis showed that female gender (odds ratio = 5.45, *p* value = 0.034), positive history of cancer (odds ratio = 5.14, *p* value = 0.044), and increased length of hospital stay (odds ratio = 1.19, *p* value < 0.001) were associated with a higher DVT incidence, whereas chemoprophylaxis use (odds ratio = 0.19, *p* value = 0.011) was associated with lower DVT incidence.

### Comparison between patients with and without chemoprophylaxis

Twenty out of 199 (10.05%) patients with only mechanical thromboprophylaxis developed DVT. In contrast, four out of 117 (3.42%) patients with additional chemoprophylaxis developed DVT. Multivariate analysis showed that chemoprophylaxis use was associated with a statistically significant reduction of DVT incidence (odds ratio = 0.19, *p* value = 0.011).

### Chemoprophylaxis use and patient-reported outcome measures (PROM)

Mann-Whitney’s U test was conducted to analyze PROM in relation to chemoprophylaxis use (Table 4). Our study found no statistically significant difference in PROM between patients that were and were not given chemoprophylaxis.

**Table 4:** Patient-reported outcome measures relative to chemoprophylaxis use

		Patients given chemoprophylaxis (median)	Patients not given chemoprophylaxis (median)	*p* value
Pre-op	SF36v2 PCS	30.45	30.09	0.312
	SF36v2 MCS	58.68	58.60	0.171
	WOMAC	67.20	65.20	0.222
	KSS Function	50.00	50.00	0.249
	KSS Knee	41.50	36.00	0.054
Post-op 6 months	SF36v2 PCS	48.53	47.94	0.857
	SF36v2 MCS	58.60	58.50	0.403
	WOMAC	91.70	90.63	0.537
	KSS Function	80.00	70.00	0.325
	KSS Knee	94.00	94.00	0.687
Post-op 24 months	SF36v2 PCS	48.00	49.37	0.391
	SF36v2 MCS	59.00	58.83	0.352
	WOMAC	90.91	92.97	0.218
	KSS Function	80.00	80.00	0.189
	KSS Knee	95.00	97.00	0.979

*SF36v2 PCS* Short Form 36 Version 2 – Physical Component Summary*, SF36v2 MCS* Short Form 36 Version 2 – Physical Component Summary*, WOMAC* Western Ontario and McMaster Universities Osteoarthritis Index*, KSS Function* Knee Society Score – Function Score*, KSS Knee* Knee Society Score – Knee Score

## Discussion

### Our results

The overall DVT incidence in our study, as diagnosed with DUS, was 7.59%. The proximal DVT incidence was 0.95%, and the distal DVT incidence was 6.65%. Only one case of symptomatic DVT was observed in our study. This finding is consistent with findings from previous studies that employed routine DUS in the detection of DVT after TKA [12, 15]. Loh et al. reported an overall DVT incidence of 4.50% (proximal 0.87%, distal 3.63%) in a study of 2978 patients. Previous studies have also shown that most DVT are asymptomatic and that classical clinical manifestations of acute-onset pain, swelling, erythema, and/or warmth of the lower extremity are neither specific nor reliable [16, 17].

Our results suggest that the use of chemoprophylaxis in addition to mechanical thromboprophylaxis might be effective in reducing DVT incidence after TKA. Multivariate analysis showed a statistically significant (*p* value = 0.019) reduction in DVT incidence between patients who received only mechanical thromboprophylaxis (10.05%) and patients who received additional chemoprophylaxis (3.42%). However, most DVT observed in our study were distal DVTs, which have limited clinical significance as they are less strongly associated with PE compared to proximal DVT [18, 19].

### What is the optimal strategy of thromboprophylaxis?

The optimal modality of thromboprophylaxis remains unclear. While most international guidelines recommend the use of thromboprophylaxis in the form of pharmacologic agents and/or mechanical compressive devices, no general consensus exists on which modality is preferred [4, 5, 20]. Although mechanical compressive devices are attractive because they do not increase bleeding, some have questioned their efficacy compared to pharmacologic agents in preventing VTE [20]. However, recent studies have shown that mechanical thromboprophylaxis and early mobilization alone might be sufficient in low-risk patients [11–13]. In a study involving 13,384 patients, Gill et al. found that mechanical thromboprophylaxis with early mobilization was just as effective as chemoprophylaxis in reducing the incidence of DVT and PE [7].

Agreement is also lacking on the ideal pharmacologic agent. While American College of Chest Physicians (ACCP) guidelines recommend the use of LMWH in preference to other pharmacologic agents due to its established track-record of safety and efficacy, American Society of Hematology (ASH) guidelines suggest using aspirin or anticoagulants because they have been shown to have comparable efficacy, albeit with aspirin having a slightly increased risk of bleeding [20, 21]. The ASH guidelines also recommend that if anticoagulants are used, direct oral anticoagulants (DOACs) are preferred over LMWH due to their slightly better efficacy in preventing PE and proximal DVT and for their similar safety profiles [21].

The optimal duration of thromboprophylaxis is also controversial. ACCP guidelines recommend thromboprophylaxis for a minimum of 10 to 14 days, extendable up to 35 days, whereas AAOS finds the current evidence inconclusive and recommends that the duration of chemoprophylaxis be decided on an individual basis by the patient and physician [5, 20]. Moreover, extended-duration chemoprophylaxis has only been shown to be effective following hip replacements but not knee replacements [22]. Enhanced recovery protocols in modern elective TKA also often involve early postoperative mobilization and shorter hospital stays, which can reduce the need for thromboprophylaxis by reducing DVT incidence [23]. Pearse et al. observed that early mobilization within 24 h after surgery was associated with a 30-fold reduction in DVT incidence [24]. Prescribing extended-duration chemoprophylaxis for patients with short hospital stays also means that patients would require post-discharge prophylaxis, with additional costs and burden on the patient and their caretakers [23, 25]. In our study, chemoprophylaxis was administered for a median duration of 4 days, until patients were ambulating and DUS confirmed the absence of DVT. The authors of this study opted for short duration, in-hospital-only chemoprophylaxis, as the general consensus was that once the patient was ambulating well, the potential bleeding risks of chemoprophylaxis may outweigh the benefits.

### Do distal DVTs matter?

The clinical significance and management of distal DVT is another area of controversy [26]. Unlike proximal DVT and PE, which have been extensively studied, with anticoagulation being the mainstay of treatment, much less is known about the optimal management of distal DVT [19, 27]. While some advocate anticoagulation to prevent proximal extension and PE, others favor a more conservative approach of close monitoring and ultrasound surveillance as distal DVT are less likely to extend to proximal veins and lead to PE [18, 19, 28]. Currently, the ACCP guidelines suggest serial imaging for low-risk patients with distal DVT and anticoagulation for high-risk or severely symptomatic patients only [27]. In our study, patients with distal DVT were either given anticoagulation or underwent close monitoring with repeat DUS scans according to their risk profile. Of note, one patient in our study with a distal DVT discovered on POD3 subsequently developed symptomatic PE on POD4 confirmed by CTPA. Although this was an isolated case, it is a reminder that not all distal DVT are completely benign. Further studies are required to better understand the clinical significance of distal DVT and their optimal management.

### Limitations

First, this is a retrospective study and thus subject to selection bias. Next, our study employed early DUS scan within 6 days after surgery to detect the presence of DVT. Although DUS has the advantages of being noninvasive and cost effective, with no risk of radiation, CT venography is the reference standard and widely regarded as a more sensitive method of detecting DVT [29, 30]. The ideal timing to perform this scan, as well as the number of scans required, remains unclear. In our study, two patients initially showed normal DUS scans on POD4 but subsequently developed DVT on POD11 and POD18, respectively. The potential benefits of repeat scans should be weighed against the costs and inconvenience for the patient, especially in a low-incidence population. Furthermore, slight variability existed in the type of chemoprophylaxis used in our study, although enoxaparin was used in the vast majority (96.6%). Lastly, the one case of PE was insufficient for meaningful analysis and significant conclusions to be drawn about chemoprophylaxis use and the incidence of PE.

## Conclusion

Our study has shown that despite the low incidence of DVT in Asian patients undergoing TKA, short duration, in-hospital-only chemoprophylaxis in addition to mechanical thromboprophylaxis might be effective in reducing the incidence of DVT. However, most DVT observed in our study were distal and may be of limited clinical significance. Further studies are needed to investigate the impact of chemoprophylaxis use on PE incidence and overall mortality rates among Asian patients.

## Data Availability

The datasets analyzed in the current study are not publicly available due to patient data confidentiality but are available from the corresponding author on reasonable request.

## Abbreviations

VTE: Venous thromboembolism
DVT: Deep vein thrombosis
PE: Pulmonary embolism
TKA: Total knee arthroplasty
DUS: Doppler ultrasound
POD: Postoperative day
CTPA: Computed tomography pulmonary angiogram
BMI: Body mass index
PROM: Patient-reported outcome measures
WOMAC: Western Ontario and McMaster Universities Osteoarthritis Index
SF-36: Short Form-36
KSS: Knee Society Score
AAOS: American Academy of Orthopedic Surgeons
NICE: National Institute for Health and Care Excellence
ACCP: American College of Chest Physicians
ASH: American Society of Hematology
